# Evaluation of Insecticidal Activity of Macrolide and Neonicotinoid Insecticides Against *Zeugodacus tau* (Walker) and Their Residue Dissipation Dynamics in *Luffa cylindrica*

**DOI:** 10.3390/insects17030242

**Published:** 2026-02-26

**Authors:** Xingyu Jia, Min Liu, Yaqian Shang, Hina Gul, Yuxi Wang, Yulu Mao, Shuxing Zhou, Tingzhe Sun, Jinming Zhang

**Affiliations:** 1 Key Laboratory of Biodiversity Conservation and Characteristic Resource Utilization in Southwest Anhui, School of Life Sciences, Anqing Normal University, Anqing 246133, China; 17856765895@163.com; 2 State Key Laboratory for Quality and Safety of Agro-Products, Key Laboratory of Biotechnology in Plant Protection of MOA of China and Zhejiang Province, Institute of Plant Protection and Microbiology, Zhejiang Academy of Agricultural Sciences, Hangzhou 310021, China3090100232@zju.edu.cn (S.Z.); 3 College of Life and Environmental Sciences, Hangzhou Normal University, Hangzhou 311121, China; 13323871140@163.com; 4 College of Agronomy, Sichuan Agricultural University, Chengdu 611130, China; maoyuluyl@163.com

**Keywords:** *Zeugodacus tau*, macrolides, neonicotinoids, developmental stage sensitivity, pesticide residue distribution, degradation dynamics

## Abstract

*Zeugodacus tau* (Walker) is a major invasive pest that targets cucurbitaceous plants and other economically significant crops, causing substantial yield and quality losses. The pest’s method of infesting the fruit, however, limits the efficacy of conventional chemical control strategies. This study comprehensively evaluates the insecticidal efficacy of seven insecticides from two key classes—macrolides and neonicotinoids—against various developmental stages of *Z. tau*. Additionally, it investigates the distribution and degradation patterns of pesticide residues on sponge gourd. Laboratory toxicity tests showed notable differences in sensitivity among the three pest stages. Larvae were the most sensitive to insecticides, followed by adults, with eggs being relatively resistant. Four macrolide insecticides showed high activity against both adults and larvae, while three neonicotinoids displayed low activity against eggs, larvae, and adults of *Z. tau*. Field residue trials using common recommended dosages demonstrated rapid degradation of pesticide residues on sponge gourd, with residue levels dropping below the national maximum residue limits (MRLs) within 2 to 24 h after application. Further results indicated that pesticide residues accumulated mainly on the peel, with low or undetectable levels in the flesh. Among the macrolide insecticides, residues were nearly undetectable, whereas neonicotinoids, due to their systemic nature, sometimes showed trace residues in the fruit flesh. Oviposition hole measurements revealed an average depth of 0.57 ± 0.29 cm, just beneath the skin, with the hole’s structure effectively protecting the eggs laid by females. Based on the comprehensive analysis of insecticide toxicity and residue degradation, control strategies should primarily target adults. Macrolide insecticides, which have low residue risks and high efficacy against both adults and larvae, are recommended as the preferred option for management. This study, considering the unique damage characteristics of *Z. tau*, offers valuable insights into chemical control strategies, focusing on insecticide sensitivity at different life stages, residue distribution, and degradation, providing important references for the scientific use of pesticides and the optimization of pest management approaches.

## Simple Summary

Sponge gourd (*Luffa cylindrica*) is a widely cultivated crop in the Cucurbitaceae family, commonly affected by *Zeugodacus tau* in southern China. Chemical control remains the main method for managing *Z. tau*; however, improper pesticide use can lead to pest resistance and high pesticide residues, posing food safety risks. Our study assessed the insecticidal activity of seven key insecticides from two major classes against different developmental stages of *Z. tau* and monitored the degradation of these pesticides on sponge gourd. Results showed that pesticide residues were mainly concentrated on the peel, with much lower levels in the flesh. Macrolide insecticides had significant effects on both adults and larvae, degrading quickly and posing low residue risks. However, their efficacy against larvae that had already penetrated the flesh was limited. These findings offer valuable guidance for farmers to select effective, low-residue pesticides and optimize pest control strategies for fruit flies.

## 1. Introduction

*Zeugodacus tau* (Walker) belongs to the order Diptera, the family Tephritidae, and the genus *Zeugodacus* [1]. This polyphagous pest is widely distributed across tropical, subtropical, and temperate regions. The species has a broad host range, encompassing up to 91 species from 16 families [2]. In addition to damaging crops from the Cucurbitaceae family—including Luffa, Cucurbita, Benincasa, Momordica, and Cucumis—it also affects economically important crops from the Rutaceae and Solanaceae families [3]. *Z. tau* shows high environmental adaptability and is commonly spread through its eggs, larvae, and pupae, which are carried by host plants and packaging [4], indicating significant invasive potential [5]. It has been classified as a major quarantine pest in many countries [6,7]. Currently, *Z. tau* has spread throughout most of southern China [3,8] and is a primary pest affecting economic crops in Zhejiang Province. The female adult lays eggs by inserting her ovipositor into the skin of the fruit, damaging the fruit or vegetables. The eggs hatch into larvae that feed on the decaying flesh of the fruit. As a result, a large portion of infected fruits or vegetables rot and fall prematurely from the plant, severely affecting the quality and yield of agricultural products [9,10]. Therefore, integrated pest management targeting multiple life stages of the pest—especially control of adult flies—is a vital strategy for reducing the damage caused by *Z. tau*.

Sponge gourd (*Luffa cylindrica*) is an important economic crop in the Cucurbitaceae family [11,12]. It is native to the tropical and subtropical regions of Asia and has become a widely grown and consumed vegetable in China due to its rich nutritional and medicinal value [13,14]. In Zhejiang Province, sponge gourd cultivation is primarily implemented on a small scale by farmers, with an annual planting area exceeding 10,000 hectares. The planting season for field-grown sponge gourd begins in mid-March for spring planting and from mid to late July for autumn planting. Sponge gourd can be harvested year-round until mid- to late October [15]. Since sponge gourd and other Cucurbitaceae crops are preferred hosts for *Z. tau* [6,10], sponge gourd is cultivated nearly year-round in Zhejiang Province, except during winter. This provides abundant host plants for the growth and development of *Z. tau*, which has caused significant infestations over the past three years, severely threatening the production of sponge gourd and other Cucurbitaceae crops [16].

To date, various pest control methods, including biological and physical control, have been used to manage *Z. tau*. However, chemical control remains the main approach for managing *Z. tau* [17,18]. The purpose of chemical control is to use insecticides not only to directly kill adults but also to target eggs laid on the skin of sponge gourd and larvae feeding inside the fruit flesh, while ensuring that pesticide residues stay within safe limits. Multiple indoor trials have shown that many insecticides are effective at killing different life stages of *Z. tau*, such as eggs, larvae, pupae, and adults [17,19,20]. However, results from field applications often do not match laboratory toxicity data, which are mainly affected by factors like the insecticide’s inherent properties (active ingredient content, adjuvants, and mode of action) [21,22,23], environmental conditions (temperature, light, and rainfall) [24], application equipment, resistance, and the pest’s specific feeding behavior [25,26].

For the effective and safe cultivation of sponge gourd, selecting efficient and low-toxicity pesticides is crucial. Our study focuses on seven pesticides: spinetoram, spinosad, emamectin benzoate, avermectin, thiamethoxam, nitenpyram, and imidacloprid; based on the efficacy and registration status of the pesticides, these seven agents are the core pesticides for controlling major pests of sponge gourd. Spinetoram and spinosad, both macrolide bioinsecticides, are highly effective against pests like thrips and diamondback moths, with low toxicity to non-target organisms [27]. Abamectin is a broad-spectrum antibiotic insecticide effective against pests such as the citrus fruit fly [28]. Thiamethoxam, acetamiprid, and imidacloprid, all neonicotinoid-based insecticides, are widely used for their broad-spectrum action and long-lasting efficacy against sap-feeding pests, despite concerns about residue persistence and resistance development [29,30]. These pesticides were selected to explore their residue degradation patterns on sponge gourd and guide safe pesticide usage.

Therefore, the study introduces a comprehensive approach to pest control by examining the insecticidal activity of macrolide and neonicotinoid insecticides at multiple developmental stages of *Z. tau* and investigating the degradation of pesticide residues on sponge gourd [24]. Previous studies have predominantly focused on the toxicity of insecticides to specific pest life stages or on residue degradation in isolation. However, our study integrates these two aspects, providing a holistic understanding of how insecticide toxicity interacts with residue dissipation over time. This integrated analysis allows us to draw more robust conclusions regarding the optimal use of pesticides, aiming to reduce pest populations while minimizing pesticide residues on the crop.

## 2. Materials and Methods

### 2.1. Materials

Information on insecticides used for laboratory bioassays is shown in Table S1. The tested compounds included neonicotinoids (thiamethoxam, nitenpyram, and imidacloprid) that target the nicotinic acetylcholine receptors, macrolides (spinetoram and spinosad) that act on nicotinic acetylcholine receptors (distinct site from neonicotinoids) and GABA receptors, and avermectins (emamectin benzoate and abamectin) that act as agonists of glutamate-gated chloride channels (GluCls) and also interfere with GABA receptors. Information on insecticides used for field pesticide residue experiments is presented in Table S2. All standards were prepared at a concentration of 50 μg/mL, as shown in Table S3.

Other reagents used for pesticide residue analysis included acetonitrile and methanol (HPLC grade; Merck, Darmstadt, Germany), formic acid, and ammonium formate (HPLC grade; Aldrich, Schnelldorf, Germany). QuEChERS extraction salt packets (containing 4 g MgSO_4_, 1 g NaCl, 1 g sodium citrate, and 0.5 g disodium hydrogen citrate) and cleanup sorbent packets [containing 150 mg anhydrous MgSO_4_ and 25 mg Primary Secondary Amine (PSA)] were purchased from Beijing Dima Technology Co., Ltd. (Beijing, China).

Instruments and accessories used for pesticide residue analysis included an H-Class TQ-S ultra-performance liquid chromatography–tandem mass spectrometry system (UPLC–MS/MS; Waters, Milford, MA, USA), a Phenomenex Polar C18 column (3.0 × 100 mm, 3 μm), a KS4000i constant-temperature shaker and a GENIUS3 vortex mixer (IKA, Staufen im Breisgau, Germany), and a 3K15 high-speed centrifuge (Sigma, Darmstadt, Germany).

### 2.2. Insects

*Zeugodacus tau* (Walker) was collected in late October 2024 from a sponge gourd planting area in Jinfan Village, Baiyang Subdistrict, Wuyi County, Jinhua City, Zhejiang Province, China (28°56′43″ N, 119°49′26″ E). Infested sponge gourds were transported to the laboratory, where *Z. tau* was reared and maintained under controlled conditions. All insects were cultured in an artificial climate chamber at 26 ± 1 °C, 60 ± 10% relative humidity, and a photoperiod of 14:10 h (L:D). Larvae collected from decayed sponge gourds were transferred to metal trays (40 cm × 25 cm) containing a 5 cm layer of fine sand. The sand was moistened with tap water to approximately 75% moisture content. Mature larvae burrowed into the sand and pupated there. Newly emerged adults were fed an artificial diet consisting of brewer’s yeast powder and sucrose, with water provided via soaked cotton wicks.

After sexual maturity, fresh zucchini (*Cucurbita pepo*) fruits were used as oviposition substrates. About 5 h later, zucchini containing eggs were transferred to plastic larval-rearing boxes (25 cm × 15 cm × 10 cm) and enclosed with nylon mesh to prevent larval mortality caused by excess moisture from fruit decay. Eggs hatched within 1–2 days, and larvae fed inside the fruit for approximately 4–5 days until reaching the mature larval stage. When larvae stopped feeding and started jumping, they were washed out and moved to pupation boxes filled with 3–5 cm of 75% moist, sterilized sand, where pupation took place.

Pupation boxes were placed in insect-rearing cages under consistent environmental conditions. Adults emerged after approximately 7–10 days and were transferred to new cages to start the next generation. The experimental population had been continuously reared under laboratory conditions for over 15 generations. We used the F20 generation of *Z. tau* for testing the insecticidal efficacy of the selected pesticides. Early generations of insects may have developed resistance due to previous exposure to insecticides. Individuals at the appropriate developmental stage and age were selected for insecticide bioassays.

### 2.3. Laboratory Bioassays of Insecticidal Activity

All seven insecticides were initially dissolved in acetone to prepare stock solutions and subsequently diluted with distilled water to obtain 5–7 tests based on preliminary assays.

#### 2.3.1. Adult Bioassays

Adult toxicity was assessed using the drug membrane contact method following [17,31,32], with minor modifications. Briefly, 5 mL of insecticide solution was added to a 250 mL Erlenmeyer flask. The flask was gently rotated to evenly coat the interior surface, after which excess solution was discarded. Once the solution had fully evaporated, 15-day-old adult *Z. tau* were introduced into the flask. A cotton ball soaked in 5% honey solution served as food, and the flask opening was sealed with nylon mesh secured by a rubber band. Flasks were placed horizontally in an artificial climate chamber under the conditions described above. For the control treatment, the flasks were coated with an acetone-in-water solution at the highest solvent concentration used in the insecticide dilutions and allowed to dry completely, thereby excluding any solvent-induced mortality. According to standard bioassay guidelines and recommended experimental designs, the number of replicates for each treatment should provide sufficient statistical power to ensure the reliability and reproducibility of the experimental results [33]. In our study, each concentration was replicated four times, with 15 adults per replicate. Mortality was recorded after 24 h. Adults were considered dead if they were unable to stand or walk normally when gently prodded with a fine brush.

#### 2.3.2. Larval Bioassays

Larval toxicity assays were conducted with reference to da Roza Harter et al. (2024) [34], with modifications. Fresh zucchini slices (approximately 2 mm thick) were immersed in insecticide solutions of different concentrations for 30 s, air-dried at room temperature, and placed into 9 cm diameter Petri dishes. Twenty second-instar larvae were then introduced into each dish. Petri dishes were maintained in the same artificial climate chamber conditions as described for adult bioassays. Mortality was assessed after 24 h, and larvae were considered dead if no response was observed upon gentle stimulation with a fine brush. Each treatment was replicated four times, and distilled water-treated zucchini slices served as controls.

#### 2.3.3. Egg Bioassays

For egg toxicity assays, 1000 μL of insecticide solution was applied to filter paper placed in a 9 cm Petri dish. Fifty eggs (<2 h old) were carefully transferred onto the treated filter paper using a fine brush, then an additional 200 μL of insecticide solution was gently dropped onto the eggs. Petri dishes were incubated under the same environmental conditions described above. After 24 h, egg hatchability and mortality of newly hatched larvae were assessed under a stereomicroscope. Each treatment included three replicates, and distilled water served as the control.

### 2.4. Field Residue Trials

#### 2.4.1. Study Site

Field residue trials of seven insecticides were carried out in a sponge gourd-growing area in Zhuwu Village, Xiachuan Town, Qujiang District, Quzhou City, Zhejiang Province, China (29°8′8″ N, 118°58′4″ E). The sponge gourd (‘Qusi No.1′) was planted at a density of approximately 5800 plants per hectare, corresponding to 29 plants per 50 m^2^ plot, with a row spacing of 1.8 m and plant spacing of 0.9 m, following the standard local agronomic practice in Quzhou, Zhejiang Province. Seedlings were raised on 5 March 2025, transplanted on 5 April 2025, and fruits were harvested continuously from early May to late October.

#### 2.4.2. Experimental Design

Eight treatments were established: (1) spinetoram at 22.5 g a.i./ha; (2) nitenpyram at 60 g a.i./ha; (3) avermectin at 8.1 g a.i./ha; (4) spinosad at 22.5 g a.i./ha; (5) imidacloprid at 37.5 g a.i./ha; (6) thiamethoxam at 37.5 g a.i./ha; (7) emamectin benzoate at 3.45 g a.i./ha; and (8) an untreated control. Each treatment included three replicate plots, each larger than 20 m^2^, separated by buffer rows.

Insecticides were applied in the afternoon of September 17. According to the NY/T 788-2018 standard [35], the sampling intervals at 2, 24, 48, and 72 h post-application were selected to simultaneously capture the first-order dissipation kinetics of the insecticides and to assess residues against safety benchmarks. The 2 h sample established the initial deposit (C_0_), while the 24 and 48 h intervals monitored the rapid early and mid-phase degradation, respectively. The 72 h sample was strategically aligned with common short pre-harvest intervals, providing critical data on residue levels nearing harvest and their compliance with maximum residue limits (MRLs). For each plot, containing at least 5 kg of mature, healthy fruits without visible pest or disease damage were collected. Samples were transported to the laboratory, where peel and flesh were separated, homogenized with a grinder, and stored until analysis.

#### 2.4.3. Sample Analysis

1. Preparation of Standard Solutions

Appropriate amounts of standard solutions of spinetoram (J and L isomers), spinosad (A and D isomers), emamectin benzoate, avermectin, thiamethoxam, nitenpyram, and imidacloprid were accurately transferred and diluted with acetonitrile to a final volume to prepare a mixed standard working solution at a concentration of 1 mg/L. This mixed solution was then diluted using a gradient dilution method to produce a series of matrix-matched standard working solutions at concentrations of 0.001, 0.002, 0.005, 0.01, 0.02, and 0.05 mg/L in sponge gourd peel and flesh matrices.

2. Sample Pretreatment

First, 10 g of homogenized sample (accurate to 0.01 g) was weighed into a 50 mL plastic centrifuge tube. Subsequently, 20 mL of acetonitrile, 4 g of anhydrous magnesium sulfate, 1 g of sodium chloride, 1 g of sodium citrate, 0.5 g of disodium hydrogen citrate, and one ceramic homogenizer bead were added. The tube was capped and vigorously shaken for 1 min, then centrifuged at a centrifugal force (RCF) of approximately 1932.71 g for 5 min. An aliquot of 1 mL of the supernatant was transferred into a plastic centrifuge tube containing 150 mg anhydrous magnesium sulfate and 25 mg PSA. The mixture was vortexed for 1 min and centrifuged again at 4200 r/min for 5 min. The final supernatant was filtered through a 0.22 μm membrane filter and analyzed via UPLC–MS/MS.

3. UPLC–MS/MS Conditions

Chromatographic separation was performed using a mobile phase consisting of (A) 2 mmol/L ammonium formate in water with 0.01% formic acid and (B) 2 mmol/L ammonium formate in methanol with 0.01% formic acid, with gradient elution as shown in Table S4. The column temperature was maintained at 35 °C, the sample manager temperature at 15 °C, and the injection volume was 10.0 μL

Mass spectrometric detection was carried out using an electrospray ionization source in positive ion mode (ESI+), operating in multiple reaction monitoring (MRM) mode. The capillary voltage was set at 3.5 kV, desolvation gas flow at 1000 L/h, cone gas flow at 150 L/h, source temperature at 150 °C, and desolvation temperature at 500 °C. Specific MRM transitions and parameters are listed in Table S5.

### 2.5. Statistical Analysis

Observed mortality data were adjusted using control mortality, and datasets with control mortality < 10% were deemed valid. Mortality rates and corrected mortality rates were calculated for each treatment. Microsoft Excel 2016 and SPSS 18.0 were employed for data processing and analysis. Toxicity regression equations, median lethal concentrations (LC_50_), 95% confidence intervals, and relative toxicity indices were estimated. LC_50_ values were determined using probit regression analysis.

Residue data were analyzed using Microsoft Excel and Origin 9.0. The dissipation kinetics of insecticides in sponge gourd were modeled with exponential decay, and the half-life was determined using the following equations:

Ct = C_0_ × e^−kt^    t_1/2_ = ln2/k

where C_0_ (mg/kg) is the initial pesticide residue concentration, Ct (mg/kg) is the residue concentration at time t, k is the first-order degradation rate constant, t is the sampling time (h), and t_1/2_ (h) is the dissipation half-life.

## 3. Results

### 3.1. Laboratory Toxicity of Seven Insecticides Against Zeugodacus tau

The toxicity of seven insecticides to adults was measured using a drug membrane contact method. The LC_50_ values for the adults in each treatment after 24 h of insecticide exposure are shown in Table 1. Based on the LC_50_ results, the adults were most sensitive to emamectin benzoate (0.293 mg/L), which had the lowest lethal concentration, followed by spinetoram (0.865 mg/L), spinosad (1.089 mg/L), thiamethoxam (8.921 mg/L), avermectin (14.478 mg/L), imidacloprid (40.984 mg/L), and nitenpyram (80.848 mg/L).

Larval toxicity was evaluated using the stomach poison method, and the LC_50_ values at 24 h are also presented in Table 1. Significant differences in susceptibility between larvae and adults were observed. Larvae were also most sensitive to emamectin benzoate (LC_50_ = 0.003 mg/L). The order of toxicity from highest to lowest was as follows: emamectin benzoate > spinosad (0.017 mg/L) > avermectin (0.108 mg/L) > imidacloprid (2.250 mg/L) > spinetoram (2.680 mg/L) > nitenpyram (15.930 mg/L) > thiamethoxam (36.438 mg/L).

The ovicidal activity of the seven insecticides was determined using a contact exposure assay. The LC_50_ values at 24 h are summarized in Table 1. Avermectin showed the highest ovicidal activity with an LC_50_ of 5.226 mg/L. Imidacloprid and emamectin benzoate exhibited moderate ovicidal toxicity, with LC_50_ values of 20.335 mg/L and 24.105 mg/L, respectively. This was followed by spinetoram, spinosad, and thiamethoxam, with LC_50_ values of 71.555, 80.683, and 90.857 mg/L, respectively. Nitenpyram showed the lowest ovicidal activity, with an LC_50_ of 119.399 mg/L.

Due to differences in testing methods and exposure conditions, comparisons between the sensitivity of different life stages were not conducted. The focus of this study is on the general efficacy of the selected insecticides in controlling *Z. tau*.

### 3.2. Residue Degradation Dynamics of Insecticides in Sponge Gourd

#### 3.2.1. Accuracy and Precision of the Analytical Method

The residue analysis method was developed in accordance with the national standard GB 23200.121-2021 [36]. In this study, the volume of the extraction solvent was doubled, as increasing the extraction volume generally enhances extraction efficiency. Additionally, the instrumental conditions specified in the standard method were optimized to ensure effective separation of all target compounds within a shorter analytical time.

Method validation involved spiking blank sponge gourd peel and flesh samples with seven pesticide standards at the limit of quantification (LOQ) level of 0.010 mg/kg, with five replicates for each treatment. The results indicated that recoveries of the seven pesticides ranged from 90.2% to 103% in sponge gourd flesh, with relative standard deviations (RSDs) of 1.3–2.8%. In sponge gourd peel, recoveries ranged from 88.7% to 102%, with RSDs of 1.8–4.1%. The acceptance criteria for recovery were set at 70–120%, in line with the guidelines specified in NY/T 788-2018. For RSD, an acceptable value was <20%, meeting the criteria for pesticide residue analysis.

**Figure 1:**
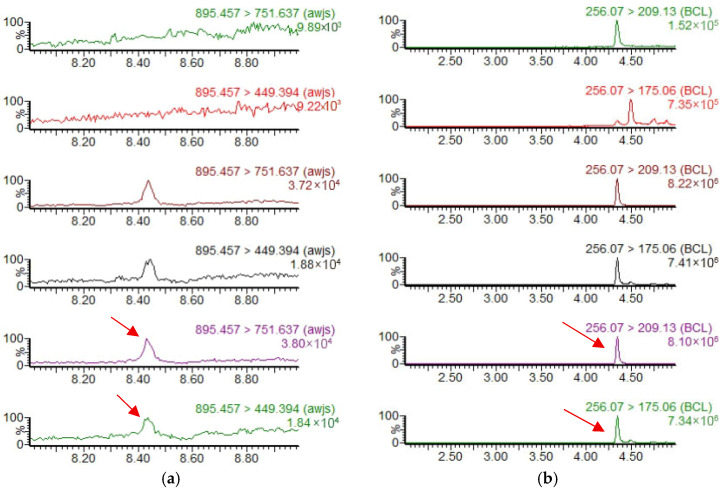

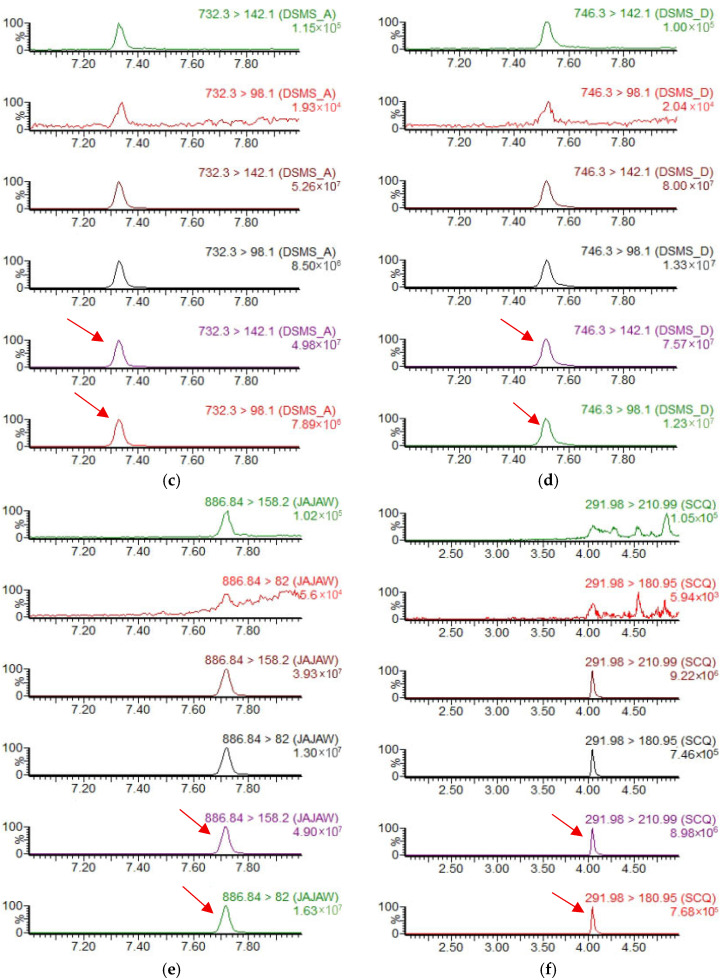

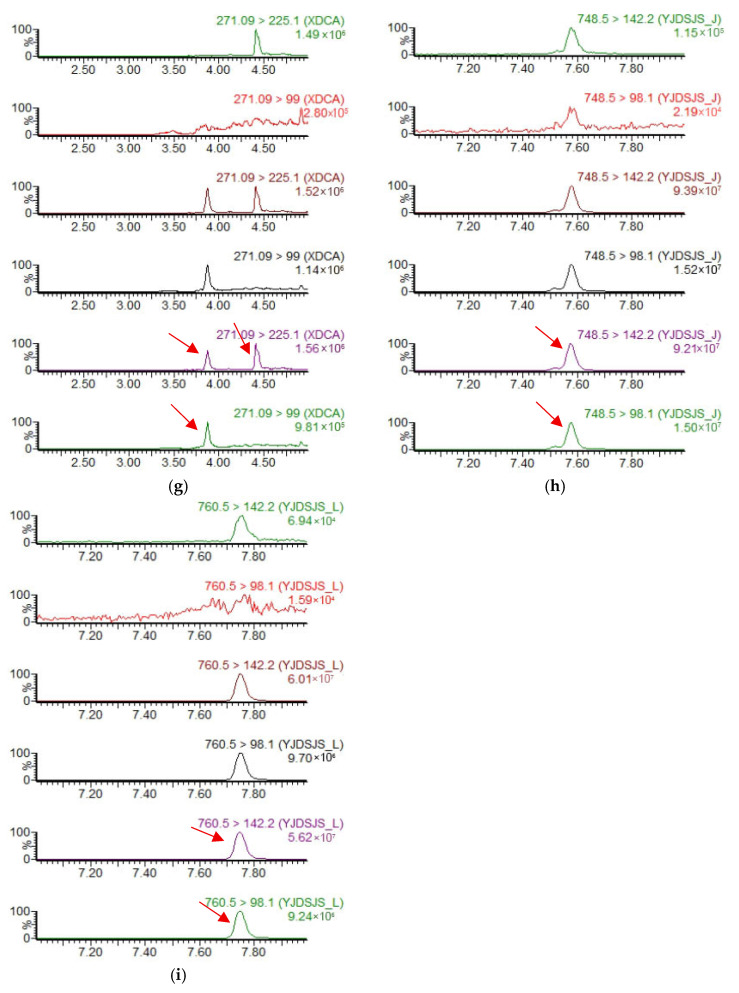
Product ion chromatograms for the validation of analytical methods for seven pesticides in fruit peel. (**a**–**i**) Product ion chromatograms of avermectin, imidacloprid, spinosad-A, spinosad-D, emamectin benzoate, thiamethoxam, nitenpyram, spinetoram-J, and spinetoram-L, respectively. From top to bottom, the chromatograms correspond to blank samples, matrix-matched standard solutions at 0.005 mg/L, and spiked samples at 0.010 mg/kg. The arrow indicates the peak of the spiked sample.

**Figure 2:**
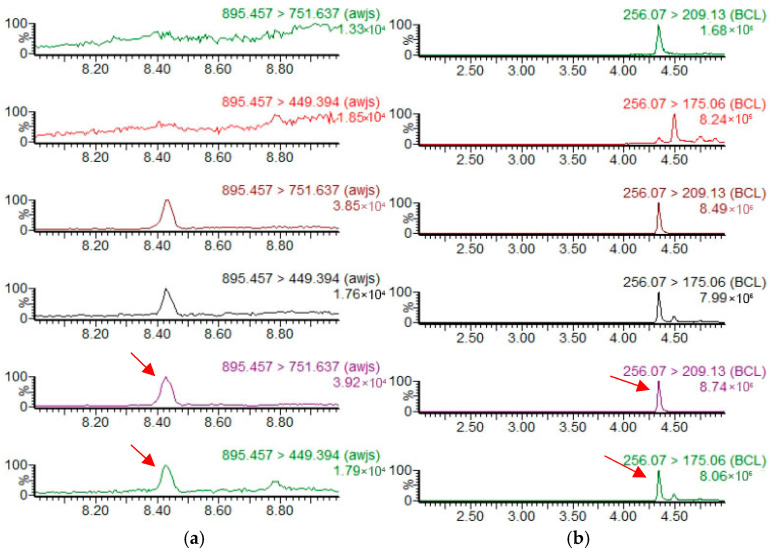

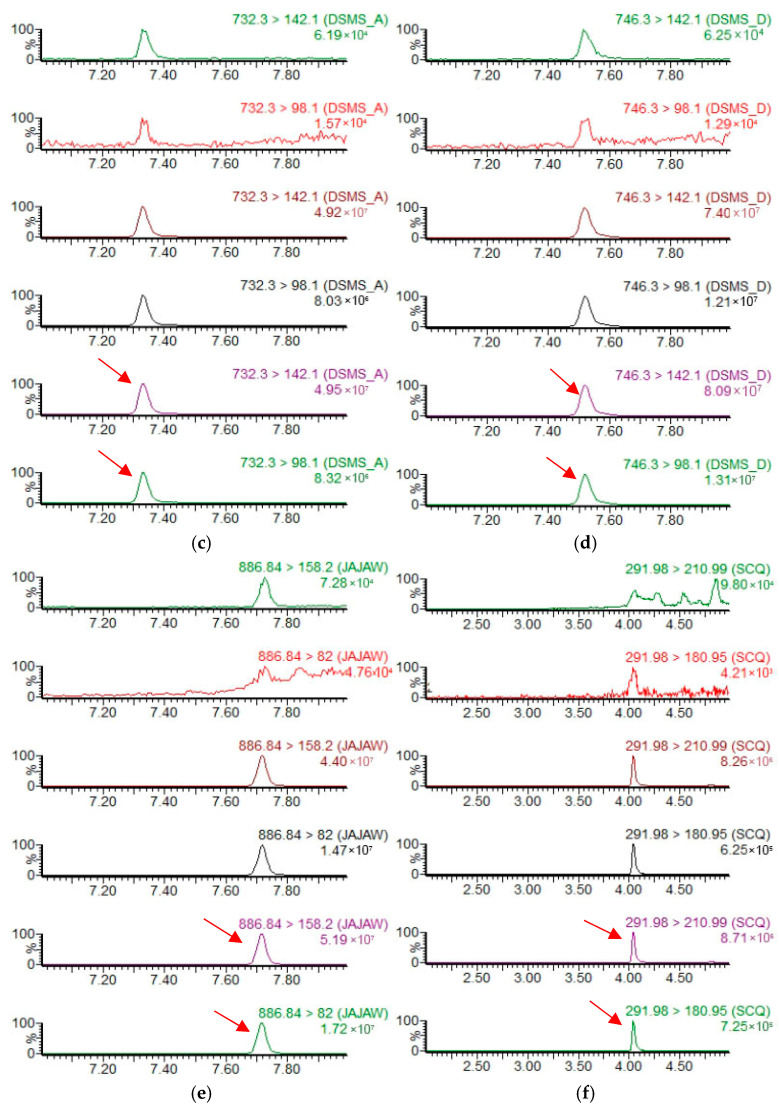

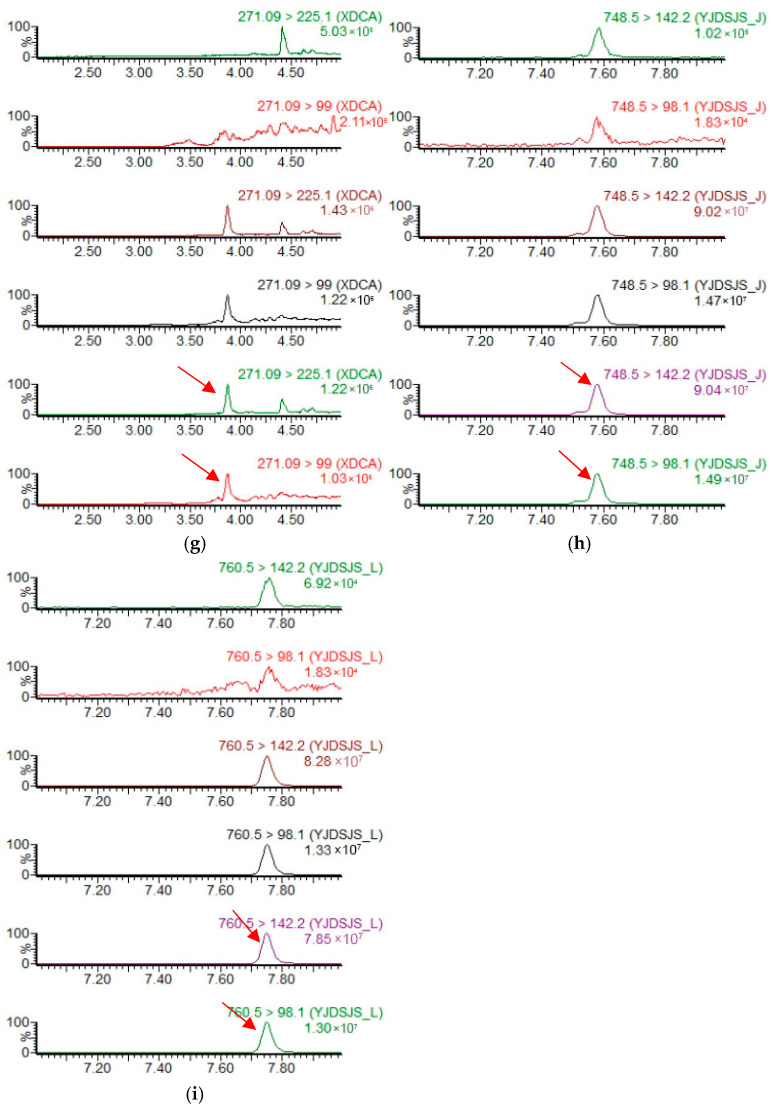
Product ion chromatograms for the validation of analytical methods for seven pesticides in fruit flesh. (**a**–**i**) Product ion chromatograms of avermectin, imidacloprid, spinosad-A, spinosad-D, emamectin benzoate, thiamethoxam, nitenpyram, spinetoram-J, and spinetoram-L, respectively. From top to bottom, the chromatograms correspond to blank samples, matrix-matched standard solutions at 0.005 mg/L, and spiked samples at 0.010 mg/kg. The arrow indicates the peak of the spiked sample.

At the LOQ spiking level, the absolute differences between the maximum and minimum measured residue concentrations ranged from 0.00031 to 0.00097 mg/kg, satisfying the precision criteria specified in GB 23200.121-2021. Representative chromatograms are shown below (Figure 1 and Figure 2).

#### 3.2.2. Residue Degradation Dynamics of Insecticides on Sponge Gourd

The adhesion and penetration of pesticides on target crops are mainly affected by pesticide properties, crop features, environmental conditions, and the use of adjuvants. During the field trial, weather conditions were favorable, with light wind and vigorous plant growth, and commonly used commercial formulations were applied. Residue analysis indicated that all seven insecticides were detectable in the peel of sponge gourd 2 h after application at recommended field rates. The mean initial deposits ranged from 0.013 to 0.153 mg/kg, with emamectin benzoate showing the lowest initial residue (0.013 mg/kg) and thiamethoxam the highest (0.153 mg/kg) (Table 2; Figure 5).

As shown in Table 2, although relatively high initial residues were found in the peel, all insecticides dissipated quickly within 24–72 h. For neonicotinoid insecticides, nitenpyram residues decreased to 16.83%, 8.9%, and became undetectable at 24, 48, and 72 h after application, respectively. Imidacloprid residues dropped to 46.5%, 37.0%, and 13.3% of the original levels, while thiamethoxam residues fell to 46.4%, 41.8%, and 14.4% over the same period. Macrolide insecticides dissipated faster than neonicotinoids. Spinosad residues decreased to 77.5% and 23.6% of the initial level at 24 and 48 h, respectively, and were not detectable at 72 h. Emamectin benzoate residues dropped to 38.5% at 24 h and were not detectable afterward. Spinetoram and avermectin dissipated most quickly, with no detectable residues in the peel at 24 h after application.

**Figure 3:**
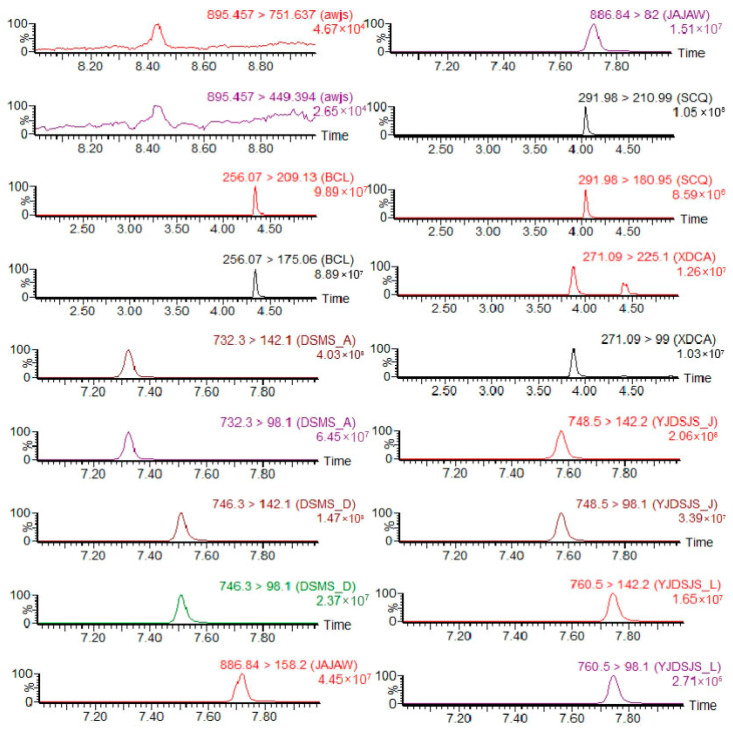
Product ion chromatograms of pesticide residues detected in sponge gourd peel samples. (From top to bottom, product ion chromatograms of avermectin, imidacloprid, spinosad-A, spinosad-D, emamectin benzoate, thiamethoxam, nitenpyram, spinetoram-J, and spinetoram-L, respectively).

**Figure 4:**
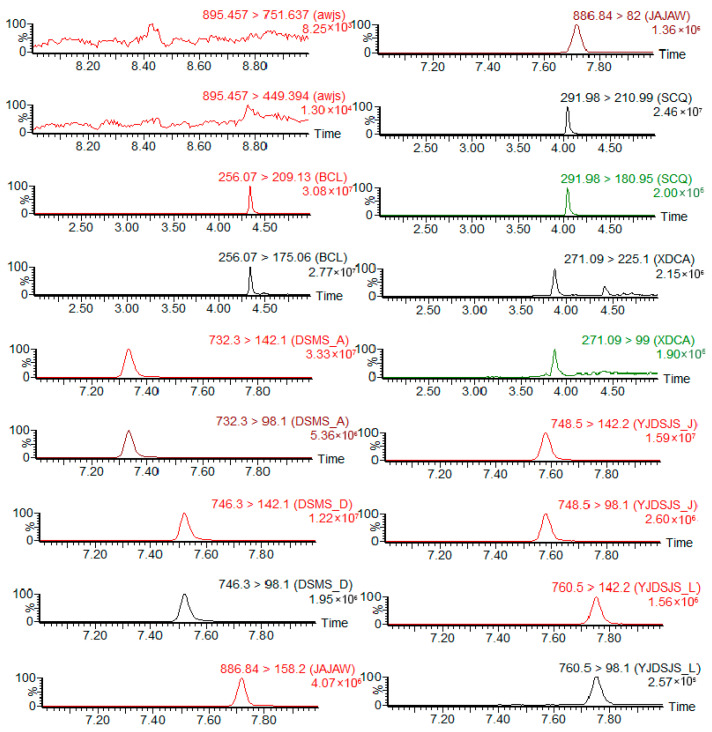
Product ion chromatograms of pesticide residues detected in sponge gourd flesh samples. (From top to bottom, product ion chromatograms of avermectin, imidacloprid, spinosad-A, spinosad-D, emamectin benzoate, thiamethoxam, nitenpyram, spinetoram-J, and spinetoram-L, respectively.).

A comparison of residue levels in the sponge gourd peel and flesh revealed that pesticide residues in the flesh were significantly lower than those in the peel. At 2 h after application, residues of emamectin benzoate, imidacloprid, avermectin, and ethyl spinosad in the flesh were below the detection limit, indicating that these insecticides either poorly penetrated from the peel into the flesh or, despite having systemic properties, were mainly confined to the peel and subepidermal tissues. Residues of the three detected insecticides—thiamethoxam, nitenpyram, and spinetoram—in the flesh measured 0.0413 mg/kg, 0.0152 mg/kg, and 0.0065 mg/kg, respectively (Table 2), representing only 26.94%, 15.05%, and 7.25% of the corresponding peel residues. The percentage of pesticide penetration into the flesh did not exceed 30%. Sample chromatograms are shown below (Figure 3 and Figure 4).

**Figure 5:**
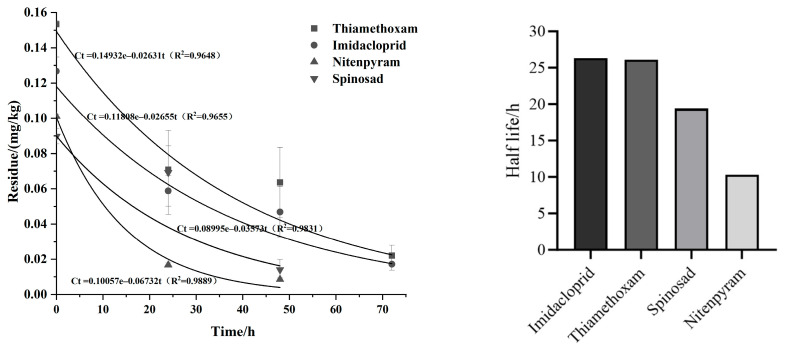
Dissipation curves and dissipation dynamics of insecticides in *Luffa cylindrica*.

Only four insecticides (thiamethoxam, imidacloprid, nitenpyram, and spinosad) provided enough residue data to model dissipation kinetics. As shown in Figure 5, residue dissipation of these insecticides in sponge gourd peel followed first-order kinetic models. However, significant differences in dissipation rates were observed among the insecticides, with half-lives ranging from 10.3 to 26.3 h. The dissipation rates ranked from fastest to slowest as follows: nitenpyram (10.3 h) > spinosad (19.4 h) > thiamethoxam (26.1 h) > imidacloprid (26.3 h).

Overall, the results show that macrolide insecticides dissipated more quickly in sponge gourd, likely because they lack systemic activity, have weaker penetration, and are more vulnerable to photodegradation. In contrast, neonicotinoid insecticides had longer half-lives, stronger systemic effects, deeper penetration into flesh tissues, and higher residue levels in sponge gourd.

## 4. Discussion

Sponge gourd is widely grown in both northern and southern China because of its easy cultivation, high nutritional value, and long harvest period, making it an important cucurbit crop. Two species of *Luffa* are found in China: sponge gourd (*Luffa cylindrica*) and ridge gourd (*L. acutangula*). In most provinces and municipalities, including Zhejiang Province, sponge gourd is the main crop, while ridge gourd is mainly grown in Guangdong, Guangxi, and Hainan Provinces [37]. Although pest complexes differ among regions due to variations in climate and geography, *Z. tau* is one of the primary pests of sponge gourd in areas with an average annual temperature above 15 °C in China [12].

The use of insecticides in crop protection remains vital for reducing pest-related crop losses and increasing overall yield [38]. Macrolide insecticides mainly work through stomach and contact toxicity. Among them, spinosyn insecticides (e.g., spinosad and spinetoram) target the insect nervous system, mainly affecting nicotinic acetylcholine receptors (nAChRs) and γ-aminobutyric acid (GABA) receptors. This dual-action approach provides high effectiveness against a variety of pests, including Lepidoptera, Thysanoptera, and Diptera and shows no cross-resistance with traditional insecticides [39,40,41]. Avermectin insecticides (e.g., avermectin and emamectin benzoate) primarily target GABA receptors, blocking signal transmission in the invertebrate central nervous system, which ultimately causes paralysis and death [42]. Neonicotinoid insecticides act primarily on the insect nervous system by targeting nicotinic acetylcholine receptors (nAChRs), which are ligand-gated ion channels involved in cholinergic neurotransmission in insects. Neonicotinoids function as agonists of insect nAChRs, binding preferentially and with high affinity to these receptors in the central nervous system, leading to persistent stimulation of nerve cells, disrupted synaptic transmission, paralysis, and eventually insect death. This selectivity for insect nAChRs over those of mammals underlies their effectiveness against sap-feeding and other insect pests and contributes to their comparatively low mammalian toxicity [29,30,43]. Neonicotinoid insecticides, known for their high toxicity to invertebrates, ease of application, systemic qualities, and long-lasting effects, are widely used to control both piercing–sucking and chewing pests. Major examples include imidacloprid, acetamiprid, thiamethoxam, dinotefuran, nitenpyram, and clothianidin, making this group one of the most popular insecticide classes worldwide [30,43]. Due to their broad-spectrum activity, high efficiency, and relatively low mammalian toxicity, both classes of insecticides are extensively used to manage *Z. tau*.

The present study demonstrated that *Z. tau* adults showed moderate sensitivity, while eggs exhibited higher tolerance. Among the seven insecticides, the LC_50_ values of emamectin benzoate, spinetoram, spinosad, thiamethoxam, nitenpyram, and clothianidin against adults were lower than the LC_50_ values against eggs. These findings align with previous toxicity evaluations of insecticides against different life stages of fruit flies [44].

Studies on pesticide residue dynamics further revealed that, after application at recommended field rates, macrolide insecticides mainly accumulated in the sponge gourd peel (including subepidermal tissues), with no detectable residues in the flesh. The pesticides are primarily concentrated in the peel. This is consistent with previous studies [45,46,47,48]. The distribution of pesticide residues in different tissues of fruits is influenced by the structure of the peel and the properties of the pesticide. For example, the waxy layer on the grape skin is thicker than that on apples, so the amount of pesticide that penetrates from the peel into the flesh of grapes is significantly lower than in apples. Systemic pesticides penetrate the peel and reach the flesh more quickly and easily than non-systemic pesticides. Pesticides with low polarity and strong lipophilicity are more likely to be retained in the waxy layer of the peel rather than penetrating into the flesh [47]. Some pesticides with high volatility may evaporate quickly and concentrate on the peel. The plant’s absorption and transport mechanisms may result in more pesticide being concentrated in the peel, with less movement into the flesh due to limitations in the plant’s transport system or the pesticide’s chemical nature. In the case of spinosad, although residues were found in the flesh, they represented only 6.7–7.2% of those in the peel, indicating limited distribution in edible tissues. Conversely, because of their systemic properties, neonicotinoid insecticides more easily penetrated into the flesh and remained for longer periods. In this study, imidacloprid was not detected in the flesh, while residues of nitenpyram and thiamethoxam found in the flesh accounted for only 12.7–59.1% of those in the peel (Table 2). Compared to the national maximum residue limits (MRLs) for pesticides in foods (Table S6), residue levels of all seven insecticides in sponge gourd sampled at 24–72 h after application at recommended doses complied fully with national food safety standards.

Based on the damage characteristics of the *Z. tau*, along with the results on pesticide residue distribution and dissipation dynamics, effective management should mainly focus on targeting adults and the eggs they lay. Previous studies in this research also measured the depth of oviposition punctures of the *Z. tau* on sponge gourd. The results showed that oviposition puncture depths ranged from 0.10 to 1.25 cm, with an average depth of 0.57 ± 0.29 cm (*n* = 22), indicating that eggs are primarily deposited in subepidermal tissues. Further dissection of sponge gourd fruits damaged by the *Z. tau* revealed that the feeding sites of mid- to late-instar larvae were mostly located in the central flesh of the fruit. This suggests that, after hatching, larvae start feeding in the subepidermal tissues; due to the large number of eggs laid and the resulting high larval density, the larvae gradually disperse and penetrate deeper into the interior of the sponge gourd (Figure S1).

A comparison of the recommended concentrations of seven insecticides with the LC_50_ values for each life stage showed that the recommended concentrations of all seven insecticides were significantly higher than the LC_50_ values for larvae but lower than or close to the LC_50_ values for eggs. Only spinosad, spinetoram, emamectin benzoate, and thiamethoxam had recommended concentrations that were much higher than the LC_50_ values for adults, meeting the control requirements for adult flies.

This study is the first to combine multi-stage insecticide susceptibility testing with the analysis of pesticide residue dissipation dynamics on sponge gourd. Unlike previous studies that have either assessed insecticide efficacy against a single life stage or focused on residue dynamics independently, our integrated approach offers a more nuanced understanding of how pesticide residues accumulate and degrade in relation to their toxicity across different life stages of *Z*. *tau*. In particular, our findings that macrolide insecticides dissipate quickly while maintaining efficacy against both adults and larvae provide valuable insights for developing pest control strategies that minimize environmental contamination. Previous research has shown that while many insecticides are effective at controlling one stage of a pest’s life cycle, they often fail to provide long-lasting control across multiple stages, especially in field conditions. In contrast, our study highlights how considering both the insecticidal activity and residue dissipation together can help optimize the timing and type of pesticide application. This holistic approach contrasts with more fragmented studies that may overlook the broader implications of pesticide use in integrated pest management strategies.

This study offers valuable insights into the chemical control of *Z. tau*. The distribution and degradation behavior of insecticides on Luffa affect pest management strategies. Controlling adults should be prioritized, and combining chemical methods with physico-chemical attractant techniques will be a key direction for *Z. tau* control. Furthermore, research on eco-friendly adjuvants that improve pesticide penetration could greatly enhance control effectiveness against eggs, which has significant research importance.

## Figures and Tables

**Table 1:** Toxicity of insecticides to *Z. tau*.

Insecticides	N	Slope (SE)	LC_50_ (mg/L) (95% CI)
**Adults**			
Emamectin Benzoate	360	2.075 (0.229)	0.293 (0.229–0.356) f
Spinetoram	360	2.172 (0.243)	0.865 (0.708–1.042) e
Spinosad	360	1.571 (0.206)	1.089 (0.859–1.399) e
Thiamethoxam	360	2.450 (0.275)	8.921 (6.937–10.820) d
Avermectin	300	2.356 (0.320)	14.478 (12.600–17.131) c
Imidacloprid	360	0.958 (0.107)	40.984 (24.907–59.491) b
Nitenpyram	540	1.990 (0.213)	80.848 (66.300–101.890) a
**Larvae**			
Emamectin Benzoate	480	1.836 (0.210)	0.003 (0.002–0.004) f
Spinosad	560	0.931 (0.158)	0.017 (0.010–0.045) e
Avermectin	640	1.063 (0.134)	0.108 (0.065–0.153) d
Imidacloprid	480	1.158 (0.183)	2.250 (1.063–3.475) c
Spinetoram	560	1.150 (0.137)	2.680 (2.067–3.542) c
Nitenpyram	480	1.763 (0.192)	15.930 (12.877–19.284) b
Thiamethoxam	560	2.798 (0.250)	36.438 (31.300–44.052) a
**Eggs**			
Avermectin	1200	2.715 (0.173)	5.226 (4.746–5.826) d
Imidacloprid	900	2.766 (0.182)	20.335 (17.982–22.658) c
Emamectin Benzoate	900	2.687 (0.186)	24.105 (21.424–26.834) c
Spinetoram	1200	1.572 (0.108)	71.555 (61.488–82.554) b
Spinosad	1050	1.907 (0.129)	80.683 (70.532–91.917) b
Thiamethoxam	1050	1.016 (0.101)	90.857 (71.939–121.165) ab
Nitenpyram	1350	1.569 (0.108)	119.399 (101.452–138.374) a

Note: N = Sample size (number of insects used per treatment group); Slope = slope of the probit regression line; SE = Standard error of the mean; LC_50_ = Lethal concentration for 50% mortality, expressed in mg/L. The statistical significance of the differences in LC_50_ values across different life stages was assessed using one-way ANOVA and independent samples *t*-tests, different letters indicate significant differences, with differences considered statistically significant at *p* < 0.05.

**Table 2:** Changes in pesticide residues (7 types) in sponge gourd flesh and peel over time.

Pesticide Type	Sampling Interval (h)	Peel (mg/kg)	Flesh (mg/kg)
Spinosad (D + A)	2	0.089 ± 0.004 a	0.006 ± 0.002 a
	24	0.069 ± 0.024 a	0.005 ± 0.000 a
	48	0.021 ± 0.006 b	-
	72	-	-
Spinetoram (L + J)	2	0.016 ± 0.006	-
	24	-	-
	48	-	-
	72	-	-
Emamectin benzoate	2	0.013 ± 0.001 a	-
	24	0.005 ± 0.003 b	-
	48	-	-
	72	-	-
Abamectin	2	0.015 ± 0.002	-
	24	-	-
	48	-	-
	72	-	-
Nitenpyram	2	0.101 ± 0.001 a	0.015 ± 0.003 a
	24	0.017 ± 0.001 b	0.003 ± 0.000 b
	48	0.009 ± 0.001 c	0.003 ± 0.001 b
	72	-	0.006 ± 0.001 c
Imidacloprid	2	0.127 ± 0.028 a	-
	24	0.059 ± 0.009 b	-
	48	0.047 ± 0.014 b	-
	72	0.017 ± 0.003 c	-
Thiamethoxam	2	0.153 ± 0.073 a	0.041 ± 0.006 a
	24	0.071 ± 0.013 a	0.009 ± 0.006 b
	48	0.064 ± 0.020 a	0.022 ± 0.012 b
	72	0.022 ± 0.006 b	0.013 ± 0.017 b

Note: “-” means not detected (ND). The statistical significance of pesticide residues was assessed using one-way ANOVA and independent samples *t*-tests, different letters indicate significant differences, with differences considered statistically significant at *p* < 0.05.

## Data Availability

The original contributions presented in this study are included in the article/Supplementary Material. Further inquiries can be directed to the corresponding authors.

## Supplementary Materials

The following supporting information can be downloaded at: https://www.mdpi.com/article/10.3390/insects17030242/s1, Figure S1: Photograph showing the oviposition hole structure of the *Z. tau* and the feeding site of its larvae on sponge gourd. Table S1: Insecticides used for laboratory experiments. Table S2: Insecticides used for field experiments. Table S3: Information on the Reference Standards for Pesticides Residue Detection. Table S4: Gradient elution program. Table S5: Multiple reaction monitoring (MRM) conditions. Table S6: National Standard for Maximum Residue Limits of 7 Insecticides in Food.
