# Supplementary material for: Evaluation of Insecticidal Activity of Macrolide and Neonicotinoid Insecticides Against Zeugodacus tau (Walker) and Their Residue Dissipation Dynamics in Luffa cylindrica
Source: Insects. 2026 Feb 26;17(3):242. doi: 10.3390/insects17030242 (PMC13027375; doi:10.3390/insects17030242)
Supplement: Supplementary file 1 [file insects-17-00242-s001.zip › insects-4146373-supplementary.pdf]

Supplementary file

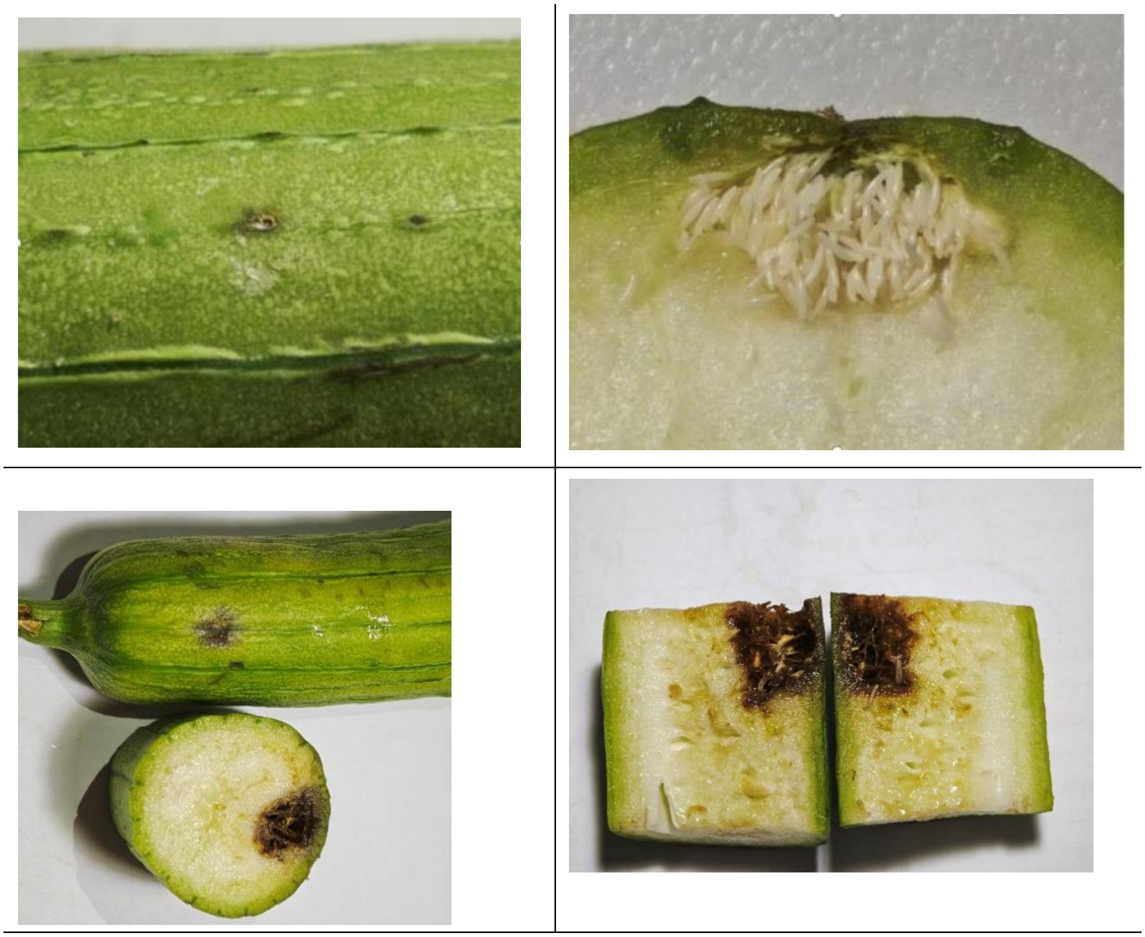

Figure S1. Photograph showing the oviposition hole structure of the *Z. tau* and the feeding site of its larvae on sponge gourd.

Table S1. Insecticides used for laboratory experiments.

Type	Common Name	Active Ingredient Content	Manufacturer
Macrolide antibiotics	Spinosad	90 %	Hangzhou Lancheng Technology Co., Ltd. (Hangzhou, China)
	Spinetoram	94 %	
	Emamectin benzonate	70 %	
	Avermectin	97 %	Shanghai Macklin Biochemical Technology Co., Ltd.
Neonicotinoid insecticide	Nitenpyram	99 %	Shanghai Macklin Biochemical Technology Co., Ltd.
	Imidacloprid	97 %	
	Thiamethoxam	95 %	Shandong Keyuan Biochemical Co., Ltd.

Table S2. Insecticides used for field experiments.

Common Name	Active Ingredient Content	Formulation	Manufacturer
Spinosad	10 %	Suspension Concentrate (SC)	Shandong LuKang Biological Pesticide Co., Ltd.
Spinetoram	60 g/L		Zhongnong Lihua Agricultural Chemicals Co., Ltd.
Emamectin benzonate	2.3 %	Microemulsion (ME)	Hebei VeYong Bio-chemical Co., Ltd.
Avermectin	1.8 %	Emulsifiable Concentrate (EC)	Hailir Pesticides and Chemicals Group Co., Ltd.
Nitenpyram	20 %	Soluble Liquid (SL)	Shandong Xinxing Pesticides Co., Ltd.
Imidacloprid	10 %	Water Dispersible Powder (WP)	Hailir Pesticides and Chemicals Group Co., Ltd.
Thiamethoxam	25 %	Water Dispersible Granule (WG)	Zhanhua Guochang Fine Chemical Co., Ltd.

Table S3. Information on the Reference Standards for Pesticides Residue Detection.

**Common Name**	**Active Ingredient Content**	**Concentration (μg/ml)**	**Manufacturer**
Spinosad	10%	50	Beijing Manhage Bio-Technology Co., Ltd.
Spinetoram	60g/L	50	
Emamectin benzonate	2.3%	50	
Avermectin	1.8%	50	
Nitenpyram	20%	50	
Imidacloprid	10%	50	
Thiamethoxam	25%	50	

Table S4. Gradient elution program

Gradient	Time(min)	Flow rate(mL/min)	Mobile phase A(%)	Mobile phase B(%)
1	0.00	0.50	97.0	3.0
2	0.20	0.50	97.0	3.0
3	1.00	0.50	90.0	10.0
4	2.50	0.50	50.0	50.0
5	4.00	0.50	30.0	70.0
6	6.50	0.50	2.0	98.0
7	8.50	0.50	2.0	98.0
8	8.60	0.50	97.0	3.0
9	11.00	0.50	97.0	3.0

Table S5. Multiple reaction monitoring (MRM) conditions

Compound	Precursor ion (m/z)	Product ion (m/z)	Dwell time (s)	Cone voltage (V)	Collision energy(eV)
Imidacloprid	256.07	175.06*	0.044	22.00	20.00
		209.13			14.00
Nitenpyram	271.09	99.00*	0.044	30.00	22.00
		225.10			14.00
Thiamethoxam	291.98	180.95*	0.044	18.00	36.00
		210.99			18.00
Spinosad-A	732.30	98.10*	0.029	30.00	70.00
		142.10			30.00
Spinosad-A	746.30	98.10*	0.029	51.00	70.00
		142.10			31.00
Spinetoram-J	748.50	98.10*	0.029	35.00	64.00
		142.20			30.00
Spinetoram-L	748.50	98.10*	0.029	35.00	66.00
		142.20			30.00
Emamectin benzonate	886.84	82.00*	0.029	48.00	55.00
		158.20			34.00
Avermectin	895.46	449.39*	0.062	92.00	26.00
		751.64			24.00

Note: *means quantification ion

Table S6. National Standard for Maximum Residue Limits of 7 Insecticides in Food

Pesticide Name	Food Category/Name	Maximum Residue Limit (MRL) (mg/kg)
Abamectin	Vegetables / Summer squash (No MRL standard specified for sponge gourd)	0.01
Imidacloprid	Vegetables / Sponge gourd	0.5
Emamectin Benzoate	Vegetables / Gourd vegetables (excluding cucumber, summer squash, and bitter gourd)	0.007
Spinetoram	Vegetables / Gourd vegetables (excluding cucumber)	0.04
Thiamethoxam	Vegetables / Sponge gourd	0.2
Spinosad	Vegetables / Gourd vegetables	0.2
Nitenpyram	Vegetables / Head cabbage (No MRL standard specified for sponge gourd)	0.2
